# Hemodynamic goals in randomized clinical trials in patients with sepsis: a systematic review of the literature

**DOI:** 10.1186/cc5948

**Published:** 2007-06-20

**Authors:** Jonathan E Sevransky, Seema Nour, Gregory M Susla, Dale M Needham, Steven Hollenberg, Peter Pronovost

**Affiliations:** 1 Department of Pulmonary/Critical Care Medicine, Johns Hopkins University, 5501 Hopkins Bayview Circle, Baltimore, MD 21224, USA; 2 Division of Cardiology, University of Wisconsin, 600 Highland Avenue H6349, Madison, WI 53792, USA; 3 MedImmune Corporation, One MedImmune Way, Gaithersburg, MD 20878, USA; 4 Division of Cardiovascular Diseases, Cooper University Hospital, Camden, NJ, 08103 USA; 5 Department of Anesthesiology/Critical Care Medicine, Johns Hopkins University, 600 North Wolfe Street, Baltimore, MD 21287, USA

## Abstract

**Introduction:**

Patients with sepsis suffer high morbidity and mortality. We sought to conduct a systematic review of the literature to evaluate the association between hemodynamic goals of therapy and patient outcomes.

**Methods:**

We conducted a comprehensive search of the literature to systematically review hemodynamic goals used in clinical trials in patients with sepsis. We searched the literature using the Pubmed (1965–June 2006), Embase (1974–June 2006), CINAHL (1982–June 2006), pre-CINAHL, and Cochrane Library (2006, issue 3) electronic databases on 1 August 2006 for the following terms: sepsis, septic shock, severe sepsis, human clinical trials. We also hand-searched references and our personal files. Studies were selected if they met all of the following criteria: randomized, controlled trial study design; enrollment of adult patients with sepsis; presence of a hemodynamic goal for patient management; > 24-hour follow-up; and survival included as an outcome. Studies were independently selected and reviewed by two investigators.

**Results:**

A total of 6,006 citations were retrieved, and 13 eligible articles were reviewed. Mean arterial pressure was a treatment goal in nine studies, and systolic blood pressure was a treatment goal in three studies. A goal for pulmonary artery occlusion pressure, central venous pressure, and cardiac index was given in four, three, and five studies, respectively. The range of hemodynamic goals used in the trials were: mean arterial pressure 60–100 mmHg, central venous pressure 6–13 mmHg, pulmonary artery occlusion pressure 13–17 mmHg, and cardiac index 3–6 l/min/m^2^. All trials that used a systolic blood pressure goal used 90 mmHg as the aim.

**Conclusion:**

For those trials that specify hemodynamic goals, the wide range of treatment targets suggest a lack of agreement on blood pressure and filling pressure goals for management of patients with sepsis. There was also inconsistency between trials in which measures were targeted. Further research is necessary to determine whether this lack of consistency in hemodynamic goals may contribute to heterogeneity in treatment effects for clinical trials of novel sepsis therapies.

## Introduction

Standard therapy for patients with septic shock includes antibiotics, infection source control, and hemodynamic support with fluids and vasoactive medications. Despite these therapies, the mortality rate for patients with sepsis remains high at 17–50% [1-3]. Recent advances in understanding the pathophysiology of sepsis have led to preclinical trials that attempted to modulate the inflammatory and coagulation pathways. Despite promising pathophysiological rationales derived from preclinical trials, most clinical trials of agents that were successful in preclinical trials did not demonstrate improved outcomes in patients with sepsis. It is unclear whether the failure to replicate the success of anti-sepsis agents seen in preclinical trials was due to the agents tested, to the hemodynamic goals of therapy chosen, or to the failure of preclinical models to reflect clinical infections.

The Surviving Sepsis Campaign published guidelines for the hemodynamic support of patients with sepsis [4]. These recommendations for treatment, however, are based primarily on expert opinion, small non-randomized trials, and short-term trials primarily aimed at demonstrating physiological principles. Hemodynamic goals vary widely among hospitals. Little is known about the variation in hemodynamic goals in clinical trials and whether this variation is associated with patient outcomes. To better understand the hemodynamic goals in clinical trials in the sepsis research, and to inform future research into anti-sepsis agents, we performed a systematic review of the literature.

## Methods

### Study selection criteria

Studies eligible for the present review met the following criteria: randomized, controlled trial study design; enrollment of adult patients with sepsis; presence of a hemodynamic goal for patient management; > 24-hour follow-up; and survival included as an outcome. The latter two requirements served to eliminate studies that were exclusively designed to measure organ function over a limited time period since this endpoint may be an inadequate surrogate for mortality in trials of novel sepsis therapies [5,6].

### Search strategy

We conducted a comprehensive search of the literature using Medline from 1 January 1965 to 1 June 2006, with the following medical subject heading terms: sepsis OR severe sepsis OR septic shock AND human clinical trials. Using similar terms, we also searched the Embase (1974–June 2006), CINAHL (1982–June 2006), pre-CINAHL, and Cochrane Library (2006, issue 3) electronic databases on 1 August 2006. We hand-searched references of relevant review articles [4,5,7] and our personal files.

### Study selection

Two investigators (JES, SN) independently reviewed citations based on the selection criteria. The abstracts of all citations selected by either of the investigators and the full-text articles for all eligible abstracts were independently reviewed by two investigators (JES, GMS). Agreement between reviewers was calculated by both the percentage agreement and kappa statistics. Disagreement regarding eligibility was resolved by consensus.

### Data extraction, synthesis, and study quality

For each eligible full-text article, two authors (JES, GMS) independently abstracted measures of patient baseline characteristics, the duration of the trial, and mortality rates. To summarize the hemodynamic goals of each trial, both measure(s) of the blood pressure and/or filling pressure used and/or the cardiac index, and the target range for each measure, were abstracted for each trial.

We evaluated study quality according to the following criteria: (1) appropriate patient selection – identification of sepsis using accepted diagnostic criteria [8], (2) control for co-interventions – standardized protocol for volume resuscitation prior to initiating vasopressors, and (3) appropriate analysis using the criteria proposed by Jadad and colleagues [9]. We used these criteria to comment on the methodological quality of studies, but did not exclude studies from the review based on this evaluation. Since the trials tested the efficacy of different sepsis agents and used different outcome measures, we could not synthesize the effect of the therapies on patient outcomes either quantitatively or qualitatively; instead, our objective was to understand the goals used for hemodynamic management of patients across these clinical trials of sepsis.

### Hemodynamic criteria

We examined the Methods sections for hemodynamic treatment goals for the clinical sepsis trials. We abstracted both treatment measures (for example, central venous pressure, mean arterial pressure (MAP)) and the hemodynamic goals of treatment (for example, central venous pressure of 8 mmHg). Hemodynamic measures and goals that were listed as part of the trial entry criteria but were not included as part of a mandated treatment strategy were excluded. We separately abstracted criteria for treatment and control groups in trials that tested specific hemodynamic endpoints. If a range of values were specified during the trial, we used the mean of the range of values specified.

## Results

**Figure 1:**
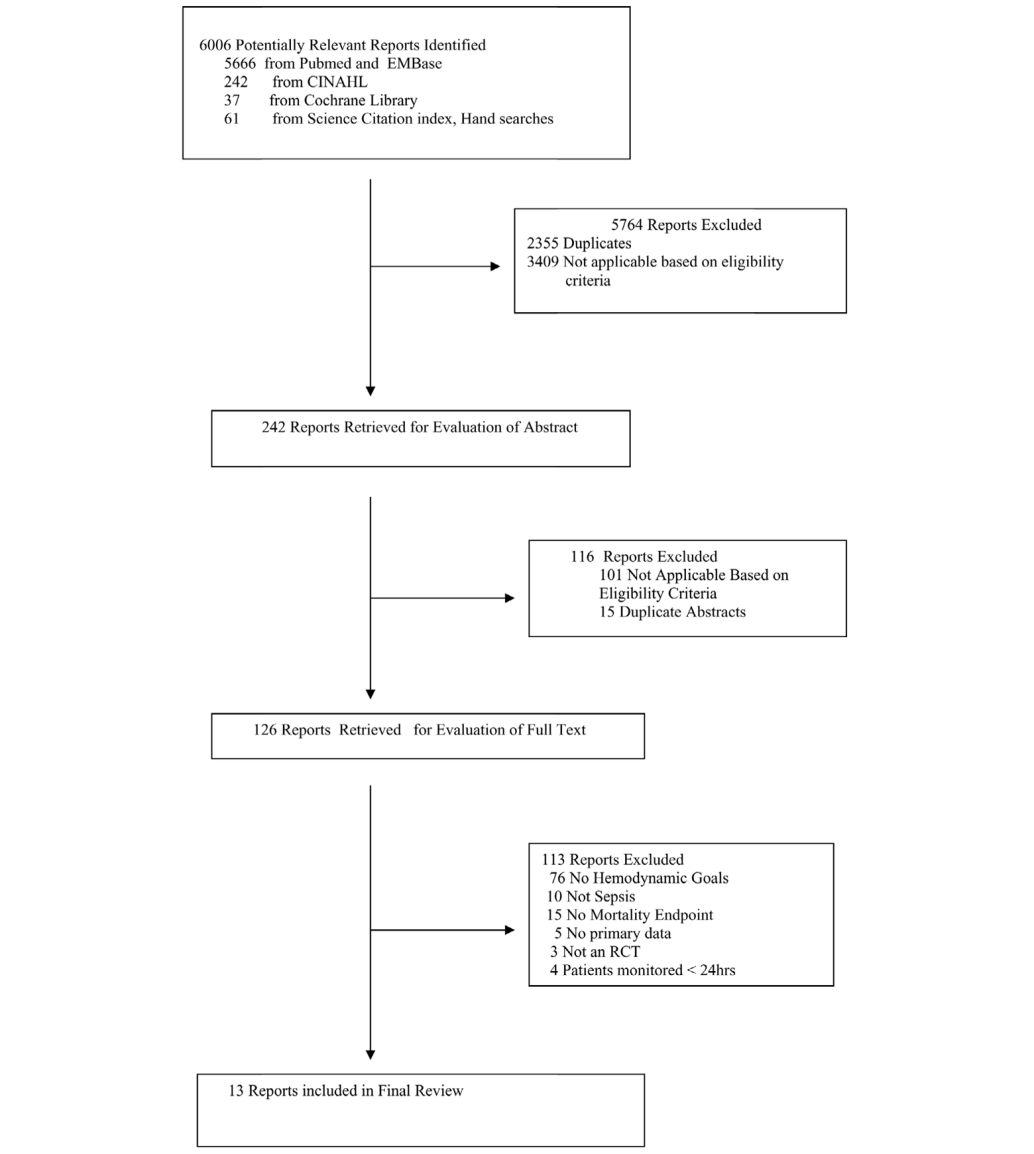
Study flow diagram. RCT, randomized controlled trial.

We identified 6,006 citations from our search strategy, of which 242 abstracts and 126 full-text articles were reviewed (Figure 1). Of these full-text articles, 10 did not enroll sepsis patients, five were secondary analysis that did not include primary data, and three were not randomized controlled trials. Of the remaining 104 studies, 76 (73%) did not include hemodynamic goals for patient management. Ultimately, 13 articles met our eligibility criteria (Table 1). Reviewer agreement on selection of eligible citations was 99% (κ = 0.79) and on selection of full-text articles was 100% (κ = 1.0).

**Table 1:** Study description

Reference	*n*	Year	Number of centers	Study population	Follow-up duration for mortality^a^
Tuchsmidt and colleagues [12]	51	1992	1	Septic shock	14 days
Peake and colleagues [10]	20	1996	1	Septic shock	Hospital stay
Bollaert and colleagues [11]	40	1998	2	Septic shock	28 days
Spapen and colleagues [19]	22	1998	1	Septic shock	Hospital stay
Alia and colleagues [18]	63	1998	1	Severe sepsis, septic shock	Intensive care unit stay
Clark and colleagues [23]	56	1998	1	Severe sepsis	40 days
Boldt and colleagues [15]	28	1999	1	Sepsis, trauma	5 days
Briegel and colleagues [17]	40	1999	1	Septic shock	365 days
Rivers and colleagues [14]	263	2001	1	Sepsis, severe sepsis, septic shock	60 days
Cole and colleagues [13]	24	2002	1	Severe sepsis, septic shock	Hospital stay
Emet and colleagues [16]	53	2004	1	Severe sepsis	Hospital stay
Bakker and colleagues [20]	312	2004	48	Septic shock	28 days
Lopez and colleagues [21]	797	2004	128	Septic shock	28 days

^a^If mortality was provided for more than one time point, the time point of the primary outcome measure was reported.

Table 3 summarizes the measures of study quality for the eligible trials. All studies reported sepsis criteria that were based on the American College of Chest Physicians/Society of Critical Care Medicine consensus criteria for entry into the clinical trial [8]. Only three studies (23%) reported a specific protocol for volume resuscitation, while 10 studies (76%) reported some measurement of organ function. Four studies (31%) met one Jadad and colleagues' criteria for study quality, six studies (46%) met two criteria, and three studies (23%) met three criteria [9].

**Table 2:** Study treatments, outcomes, and hemodynamic measurements

Reference	Treatment	*n*	Control group mortality	Study group mortality	Blood pressure goal	Other hemodynamic goals
Tuchsmidt and colleagues [12]	Elevation of cardiac output with dobutamine and fluids	51	18/25 (72%)	13/26 (50%)	SBP > 90 mmHg	Treatment group: PAOP ≥ 15 mmHg and CI ≥ 6 l/min/m^2^; control group: CI ≥ 3 l/min/m^2^
Peake and colleagues [10]	*N*-acetyl-cysteine	20	5/10 (50%)	9/10 (90%)	SBP > 90 mmHg	CI ≥ 4 l/min/m^2^; PAOP 15–18 mmHg
Bollaert and colleagues [11]	Supraphysiologic hydrocortisone	40	12/19 (63%)	7/21 (32%)	SBP > 90 mmHg	
Spapen and colleagues [19]	*N*-acetyl-cysteine	22	4/10 (40%)	5/12 (41.6%)	MAP > 65 mmHg	CI > 4 l/min/m^2^
Alia and colleagues [18]	Maximizing of oxygen delivery with dobutamine	63	21/32 (65.6%)	23/31 (74.5%)	MAP > 60 mmHg	PAOP 12–15 mmHg; treatment group: DO_2_I > 600 ml/min/m^2^; control group: DO_2_I > 330 ml/min/m^2^
Boldt and colleagues [15]	Heparin	56	11/28 (39.2%)	10/28 (35.7%)	MAP > 65 mmHg	CVP 12–15 mmHg
Clark and colleagues [23]	TNF-α antibody	28	3/14 (21.4%)	3/14 (21.4%)	MAP 90–110 mmHg	
Briegel and colleagues [17]	Stress dose hydrocortisone	40	6/20 (30%)	5/20 (25%)	MAP > 70 mmHg	PAOP 12–15 mmHg
Rivers and colleagues [14]	Multifaceted early goal-directed therapy protocol	263	70/133 (52.6)	50/130 (38.4)	MAP ≥ 65 mmHg	CVP 8–12 mmHg, EGDT SVO_2 _≥ 70%
Cole and colleagues [13]	Continuous hemofiltration	24	4/12 (33.3%)	4/12 (33.3%)	MAP ≥ 70 mmHg	
Emet and colleagues [16]	*N*-acetyl-cysteine	53	8/26 (30.7%)	7/27 (25.9%)		CVP 4–8 mmHg
Bakker and colleagues [20]	Nitric oxide synthase inhibitor	312	75/155 (48.3%)	72/155 (46.2%)	MAP ≥ 70 mmHg	CI ≥ 3 l/min/m^2^
Lopez and colleagues [21]	Nitric oxide synthase inhibitor	797	174/358 (48.6%)	259/439 (59%)	MAP 70–90 mmHg	CI ≥ 3 l/min/m^2^

CI, cardiac index; CVP, central venous pressure; DO_2_I, Oxygen Delivery Index; EGDT early goal-directed therapy SVO_2_, venous oxygen saturation; MAP, mean arterial pressure; PAOP, pulmonary artery occlusion pressure; SBP, systolic blood pressure.

**Figure 2:**
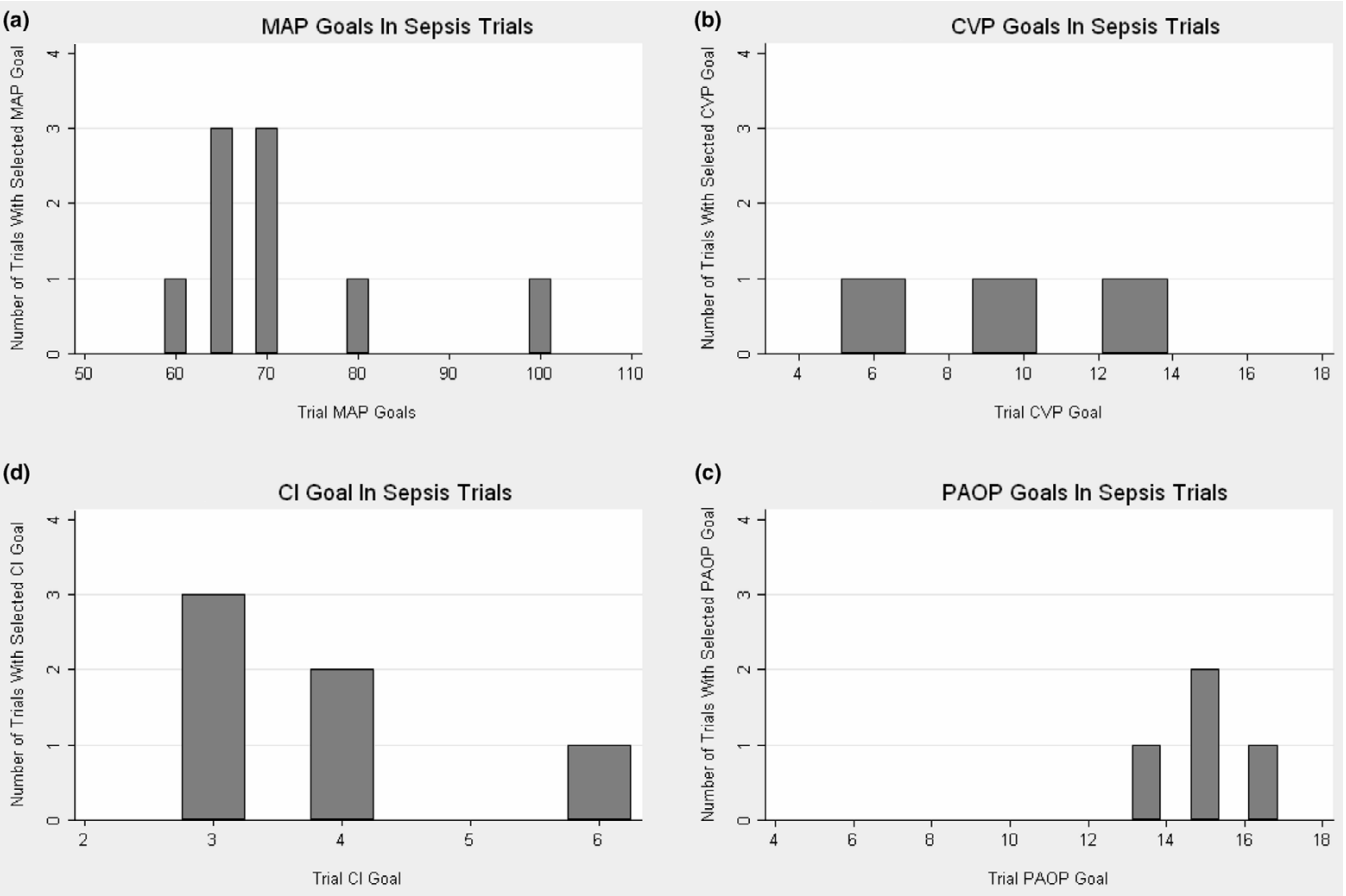
Hemodynamic goals in sepsis trials. **(a) **Mean arterial pressure (MAP) goals in sepsis trials. **(b) **Central venous pressure (CVP) goals in sepsis trials. **(c) **Pulmonary artery occlusion pressure (PAOP) goals in sepsis trials. **(d) **Cardiac index in sepsis trials. For studies that provided an interval goal range, the mean of the range is graphed. One study provided a separate CI for the treatment and control groups; these are graphed separately.

For blood pressure goals, nine studies (69%) included MAP goals, with the minimum MAP and maximum target MAP ranging from 60 to 100 mmHg (Table 2). Seven of these studies (54%) used MAP goals that fell within the range of 60–70 mmHg (Figure 2a), with the remaining two studies using 80 and 100 mmHg [10,11]. Three studies (23%) used a systolic blood pressure goal, with all studies targeting > 90 mmHg [10-12]. One study did not include any blood pressure goal [13].

For filling pressure goals, a central venous pressure goal was used in three studies (23%) [14-16] (see Figure 2b), with target goals that ranged from 6 to 13.5 mmHg. A pulmonary artery occlusion pressure goal was used in four studies (31%), with the target ranging from 13 to 17 mmHg [10,12,17,18] (see Figure 2c). A cardiac index goal was listed in five studies (38%) [12,16,19-21], with the target ranging from 3 to 6 l/min/m^2 ^(Figure 2d). One study used separate hemodynamic goals for the treatment and control arms [12]. Another study specified oxygen delivery goals [18]. In all, eight studies (61%) required a pulmonary artery catheter as part of the study procedures. Of note, one of these studies that required the use of a pulmonary artery catheter as part of the protocol did not specify treatment goals that would require the use of the catheter [11].

Three studies in the present review were designed to test specific hemodynamic treatment paradigms. Rivers and colleagues demonstrated that early goal-directed therapy over a six hour period resulted in a 12.6% absolute decrease in 60-day mortality for patients with severe sepsis [14]. Alia and colleagues examined the role of goal-directed therapy in patients with established severe sepsis and septic shock [18], and Tuchschmidt and colleagues examined the role of goal-directed therapy in septic shock [12]. The studies of both Tuchschmidt and Alia and colleagues included a treatment arm that specified supranormal therapeutic goals [12,18]. The other 10 studies incorporated specific hemodynamic goals into trials of novel therapies specifically directed at the pathophysiology of sepsis. Analysis of the studies excluding the two trials that include supranormal therapeutic goals does not alter the variability in treatment goals seen in the present review, with the exception of a narrowed cardiac index range (data not shown).

## Discussion

The present systematic review of hemodynamic goals in sepsis clinical trials has two major findings. First, of the 126 clinical studies that were reviewed in full, 73% did not include hemodynamic goals of therapy. Of the 13 studies that met our inclusion criteria, there was a wide range of targeted hemodynamic goals and measures. Importantly, not all studies included similar targets or measures.

Most of the studies used MAP as their hemodynamic measure for directing sepsis therapy. Only three of the studies used systolic blood pressure as a measure, with all three selecting 90 mmHg as the target [10-12]. While the American College of Chest Physicians/Society of Critical Care Medicine consensus definition uses systolic blood pressure as a marker of hypotension [8], some experts suggest that the MAP may be more closely associated with organ perfusion [22]. The choice of different measures in these studies may reflect variation in practice between clinicians in blood pressure targets for patients with sepsis.

In two of the studies, the MAP goal was higher than in the other studies. First, in a trial of a nonspecific nitric oxide synthase inhibitor the target MAP was between 70 and 90 mmHg, with an actual mean MAP of 86 mmHg achieved in both the treatment and control groups [21]. This trial was the first sepsis trial to demonstrate a statistically significant result, with an increase in the mortality rate for the treatment (versus placebo) group. Second, a trial of a chimeric monoclonal antibody to TNF-α targeted a MAP of between 90 and 110 mmHg [23]. In this trial there was no difference in mortality rates between the study groups. The differing results in the these two trials may have been caused by differing sample sizes of the trials, differing agents used, or other unmeasured co-interventions. Achieving a higher MAP may lower cardiac output, oxygen delivery, and regional perfusion, thus modifying the effects of sepsis therapies.

Only 54% of the studies provided a filling pressure goal as part of the treatment regimen. Three studies mandated central venous pressure goals while four studies mandated a pulmonary artery occlusion pressure goal. Adequate volume resuscitation is an essential part of hemodynamic management. While some recent studies have cast doubt on whether the pulmonary artery occlusion pressure represents an adequate surrogate for left ventricular end-diastolic volume or whether use of the pulmonary artery catheter can improve outcomes in patients with sepsis [24,25], the wide range of treatment goals and measures and the absence of a filling pressure goal in the majority of studies suggests heterogeneity in thought as regards filling pressure targets in patients with sepsis. Similar heterogeneity is seen in the cardiac index goals in the studies that included such goals.

Given the past and present interest in goal-directed therapy for patients with sepsis, we had hypothesized that a greater number of studies would be eligible for this review. Rivers and colleagues demonstrated that early goal-directed therapy over a 6-hour period for patients with severe sepsis that started in the emergency room improved outcomes [14]. It is notable that this study, in contrast to previous studies, used central venous oxygen saturation as compared with the cardiac output and mixed venous oxygen saturation measurements. Many of these studies that did not meet our inclusion criteria, however, enrolled patients who did not have sepsis but only were at risk for sepsis [26]. Furthermore, only a few studies of specific agents aimed at modulating the inflammatory cascade included specific hemodynamic goals. It is noteworthy that the four largest clinical studies evaluating novel therapies in patients with sepsis – evaluating drotrecogin alpha, tissue factor pathway inhibitor, antithrombin III, and monoclonal antibodies to TNF [27-30] – did not specify hemodynamic goals.

Only three studies included specific fluid challenge as part of their protocol [10,12,14]. All three included specific volume challenge boluses to reach a desired filling pressure, but all included different fluid-dosing and filling pressure goals. Adequate volume resuscitation remains a key component in the treatment of septic patients. While the filling pressure may represent a measure of the adequacy of resuscitation, a recent report suggests that filling pressure goals alone do not correlate well with changes in the stroke volume index [31].

The present systematic review has several potential limitations. First, the heterogeneity of populations and therapies prevents synthesis of findings regarding the hemodynamic goals on treatment outcomes It may not be possible to generalize information about treatment paradigms across these differing studies with agents with variable mechanisms of actions. The variation in treatment goals seen across these studies, however, provides evidence that practice patterns remain heterogeneous in the provision of hemodynamic support. Standardized treatment protocols have been implemented in recent years in critically ill populations, including include standard ventilatory weaning methods [32], protocolized ventilatory strategies for patients with acute lung injury [33,34], and insulin therapy goals [35,36]. Broad use of protocols to achieve hemodynamic goals in patients with sepsis, however, remains elusive.

Second, we did not include studies of patients who were at risk for developing sepsis. We therefore cannot extrapolate our findings to the general critically ill population. It is possible that those studies of the 'at-risk population' would lead to important information about the use of hemodynamic goals in critically ill populations. However, our study does provide information on those patients with established sepsis. We chose to focus on patients with sepsis since adequate supportive care with fluid and vasopressors remains one of the main tenets of therapy for patients with sepsis.

The wide range of hemodynamic goals in the selected studies underscores the lack of convincing data to support one hemodynamic goal over another, but raises the possibility that these goals may modify treatment effects of specific agents. Hemodynamic therapy is a vital portion of the treatment strategy, and it remains biologically plausible that agents affecting blood pressure and cardiac output may modify the effects of specific anti-sepsis agents. The choice of vasopressor agents for patients with septic shock may also modify the effects of such anti-sepsis agents.

The lack of specific hemodynamic measures and goals observed in the present systematic review may reflect the variation in clinicians' general beliefs and practice, or may reflect differences in patient populations studied. The heterogeneous patient population that develops sepsis (for example, elderly patient with urosepsis, young trauma patient with intraabdominal sepsis, brain-injured patient with ventilated-acquired pneumonia), however, may preclude the use of a single hemodynamic goal for all septic patients. The recently published surviving sepsis campaign guidelines do provide basic guidelines for resuscitation goals, but they suggest that the treatment goals may be individualized based on patient response to therapy [4].

## Conclusion

Fewer than 30% of all clinical trials in the field of sepsis have mandated hemodynamic treatment goals for patient management. For those studies that do report hemodynamic goals of therapy, there are wide variations in the measures followed and the goals chosen. If hemodynamic goals are related to outcomes and to specific agents, the variation in hemodynamic goals may introduce bias into clinical trials in sepsis patients. Further research is needed to determine whether standardization of measures and target goals for hemodynamic monitoring may improve clinical research in the field of sepsis.

## Key messages

• Most sepsis clinical trials reviewed did not include hemodynamic goals of therapy. Of note, the four largest clinical trials evaluating novel therapies in patients with sepsis did not specify hemodynamic goals of treatment.

• For those 13 studies identified in our systematic review, there was wide variation in hemodynamic measures selected and the hemodynamic goals chosen.

• Further research is necessary to determine whether this lack of consistency in hemodynamic goals may contribute to heterogeneity in treatment effects for clinical trials of novel sepsis therapies.

## Abbreviations

MAP = mean arterial pressure; TNF = tumor necrosis factor.

## Competing interests

The authors declare that they have no competing interests.

## Authors' contributions

All authors made a substantial contribution to the study design and methods. JES, SN, and PP planned the study. JES, SN, and GMS performed the literature review. JES, SN, DMN, and SH performed the data analysis. JES drafted the manuscript and all other authors critically revised it for important intellectual content. All authors approved the final version of the manuscript for publication.

**Table 3:** Quality assessment of trials

Reference	Sepsis criteria explicitly stated^a^	Volume challenge explicitly stated	Jadad and colleagues [9] score analysis
Tuchsmidt and colleagues [12]	Yes	Yes^b^	1
Peake and colleagues [10]	Yes	Yes^c^	2
Bollaert and colleagues [11]	Yes	No	2
Spapen and colleagues [19]	Yes	No	2
Alia and colleagues [18]	Yes	No	1
Boldt and colleagues [15]	Yes	No	1
Clark and colleagues [23]	Yes	No	3
Briegel and colleagues [17]	Yes	No	2
Rivers and colleagues [14]	Yes	Yes^d^	3
Cole and colleagues [13]	Yes	No	1
Emet and colleagues [16]	Yes	No	2
Bakker and colleagues [20]	Yes	No	3
Lopez and colleagues [21]	Yes	No	2

^a^American College of Chest Physicians/Society of Critical Care Medicine criteria [8]. ^b^5% albumin in aliquots to achieve pulmonary artery occlusion pressure > 15 mmHg. ^c^200 ml bolus over 15 minutes to achieve a sustained increase in pulmonary artery occlusion pressure ≥ 3 mmHg. ^d^20–30 ml/kg initial fluid bolus over 1 hour followed by 500 ml every 30 minutes to achieve central venous pressure of 8–12 mmHg.
